# Osteoporosis-osteopenia syndrome in children with transfusion dependent thalassemia

**DOI:** 10.1186/1753-6561-9-S7-A17

**Published:** 2015-10-27

**Authors:** Zhi-Miin Ong, Wai Cheng Foong, Seoh Leng Yeoh, Angeline Aing Chee Yeoh

**Affiliations:** 1 Royal College of Surgeons in Ireland, Dublin, Ireland; 2 Department of Pediatrics, Penang Medical College, Penang, Malaysia; 3 Department of Pediatrics, Hospital Pulau Pinang, Penang, Malaysia

## Background

Osteoporosis-osteopenia syndrome (OOS) is found in more than 50% of thalassemia patients worldwide [1,2]. Despite appropriate management, thalassemics continue to exhibit a decrease in bone mass due to the multifactorial pathogenesis of OOS in thalassemia [3]. Therefore, this study aims to obtain an insight on the thalassemia patients' perception of OOS and possible lifestyle contributing factors.

## Methods

64 regularly transfused patients from 2 major Thalassaemia Clinics in Penang aged 10 years and above participated in a 6-week cross-sectional study using a self-administered questionnaire. The questionnaire addressed lifestyle factors (exercise, diet), awareness and symptoms of bone problems. Bone profile information (serum levels of calcium, phosphate, alkaline phosphatase (ALP), 25-hydroxyvitamin D (VitD), and Bone Mineral Density T-scores [BMD]) from patients' records was documented. OOS was considered based on either BMD (lumbar and femur) of <-1.0 and/or VitD of <30ng/ml. Odds ratio was used to compare the relationship between patients' understanding of OOS and presence of symptomatic bone problems. A p-value of <0.05 was taken as significant.

## Results

25% of patients had good understanding of OOS in thalassemia. 51.6% were symptomatic, namely 20.3% had history of bone pain or fracture and 42.2% had heights below the 5th percentile. 81.2% exercised frequently and 65.6% consumed low intake of dairy products.

Only 64% of patients had either one or both BMD and VitD results. Of the available results, 87.5% had low VitD and 86.2% had low BMD. Sample serum calcium and phosphate levels were normal while the mean ALP was 164mmol/l. A better understanding on OOS reduced the odds of having symptomatic bone problems, however this was not significant (OR=0.92; p>0.05).

## Conclusion

Low-level VitD and BMD results were detected in over 80% of patients who had undergone an OOS diagnostic investigation. This is consistent with the current literature [1,2]. Many were unaware of OOS and the importance of dairy intake. This suggests that OOS is not well known. The results of this study were limited to the patient's ability to answer the questionnaire and the design of the questionnaire. T-scores were reported instead of Z-scores, which would give a better reflection of OOS in adolescents [4]. ALP could also be affected by other factors [5,6]. This study has shown the presence of OOS and its risk among the Penang adolescent thalassemia patients. Some were symptomatic. Many were unaware of OOS and took minimal dietary precaution. This calls for better education about OOS, its detection and prevention for thalassemia patients and health-care workers.
